# A case of acute myeloid leukemia-M2 with trisomy 4 in addition to t(8;21)

**DOI:** 10.4103/0971-6866.42323

**Published:** 2008

**Authors:** P. J. Trivedi, P. S. Patel, M. M Brahmbhatt, B. P. Patel, S. B. Gajjar, R. R. Iyer, E. H. Parikh, S. N. Shukla, P. M. Shah, S. R. Bakshi

**Affiliations:** Cell Biology Division, The Gujarat Cancer and Research Institute, NCH Campus, Asarwa, Ahmedabad - 380 016, India; 1 Biochemistry Research Division, The Gujarat Cancer and Research Institute, NCH Campus, Asarwa, Ahmedabad - 380 016, India; 2 Department of Medical Oncology, The Gujarat Cancer and Research Institute, NCH Campus, Asarwa, Ahmedabad - 380 016, India

**Keywords:** Acute myeloid leukemia, cytogenetics, fluorescence *in situ* hybridization

## Abstract

t(8;21)(q22;q22) is the most frequently observed karyotypic abnormality associated with acute myeloid leukemia (AML), specifically in FAB-M2. Short-term unstimulated bone marrow (BM) and peripheral blood lymphocyte culture showed 47,XX, +4,t(8;21) in all metaphase plates; and interphase and metaphase results of *AML-ETO* fusion was positive and trisomy of 4 was confirmed with WCP probes. Trisomy 4 in AML with t(8;21) is a rare numerical abnormality. Here we present such case of patient which may constitute a distinctive subtype.

## Introduction

t(8;21)(q22;q22) is the most frequently observed karyotypic abnormality associated with acute myeloid leukemia (AML), specifically in FAB M2. Trisomy 4 as the sole anomaly is a rare chromosomal abnormality associated with a specific subtype of primary acute non lymphocytic leukemia (ANLL) and secondary (treatment-related) ANLL with myelomonocytic morphology; it has been found with the same frequencies in the M1, M2, and M4 FAB phenotypes.[1,2] A retrospective analysis on the clinical and laboratory data of 21 cases of acute leukemia (AL) with trisomy 4 was performed by Pan *et al*. and showed that AL with trisomy 4 have unique clinical and laboratory features and a poor prognosis. M2 was the most frequent subtype in this series (9 out of 21 cases).[3] Association of +4 with double minute chromosomes has been described in 10 cases; 5 with AML-M2, 2 with AML-M4, 1 with refractory anemia with excess of blasts in transformation (RAEB-T), one with chronic myelomonocytic leukemia (CMMoL), and one with unclassified preleukemia. The coincidence of +4 with t(8;21) or its variant t(6;21;8) has been observed in at least two cases of ANLL (M1 and M2), is therefore recurrent. Apparently, trisomy 4 has no prognostic sifgnificance in ANLL; with the exception of the cases bearing c-kit mutations that are associated with a rapid disease progression. Trisomy 4 has been described in two cases of T-cell acute lymphoblastic leukemia as the sole chromosomal anomaly. Combined trisomies of chromosomes 4 and 10 are reported in children with B-progenitor cell acute lymphocytic leukemia and has shown a favorable prognostic association. Patients with chromosomes 4 or 10 trisomies as a sole anomaly have an extremely favorable 44-year event free survival after antimetabolite-based chemotherapy.[4]

## Case Report

A 60-year-old female with complaints of general weakness was registered at Gujarat Cancer and Research Institute in May 2007. Peripheral blood report was hemoglobin concentration 7.8 g/dL, White Blood Cell count 0.03 × 10^9^/L, polymorphs 7%, lymphocytes 14%, myelocytes 4%, blast cells 75%, and Platelet count 0.0046 × 10^9^/L. Peripheral blood smear showed presence of blasts with Auer rods. Bone marrow report revealed hypercellular marrow with marked depletion of all the normal marrow precursor cells. Few blasts showed presence of Auer rods in cytoplasm, Sudan Black-B was positive, and periodic acid Schiff (PAS) was negative, M:E ratio was altered, and megakaryocytes were not seen, lymphocytes 8%, eosinophills 1%, polymorphs 3%, band cells 2%, metamyelocytes 1%, myelocyte 8%, promyelocytes 3%, and blast cells 72%. Final diagnosis based on morphological and cytochemistry findings was AML with M2 subtype as per French-American-British classification. Constitutional nature of trisomy 4 could not be ruled out. After 1 month of sample received, the patient was lost to follow up.

### Chromosome preparation

A G-banded chromosome study was performed using standard cytogenetic protocol. Briefly, unstimulated cultures of bone marrow aspirate were set up in RPMI-1640 medium supplemented with 20% newborn calf serum, l-glutamine, and antibiotics (penicillin and streptomycin). The cells were cultured for 24 and 48 h in 5% CO_2_ incubator. Following overnight incubation in presence of Colcemid (10 µL/8 mL of culture) the cultures were exposed to hypotonic solution (0.075 mol/L KCl) and fixed with methanol:acetic acid (3:1). The slides were prepared by air-dry method and stained with GTG-banding. Twenty metaphases were analyzed and karyograms were prepared using the Cytovision computer-assisted karyotyping system (Applied Imaging, NewCastle Upon Tyne, UK). The karyotypes were described according to the International System for Human Cytogenetics Nomenclature 2005.[5]

### Fluorescence in situ hybridization (FISH) assay

FISH procedure was performed on interphase and metaphase cells following the manufacturer's (Abbott Molecular, Inc., Des Plaines, IL, USA) guidelines. The LSI *AML-ETO* dual-color dual-fusion probe was used to determine the *AML-ETO* fusion status to confirm the diagnosis of AML-M2 subtype. Whole chromosome paint for chromosome 4 with spectrum orange was applied to confirm trisomy and/or cryptic rearrangements of chromosome 4.

### Chromosome analysis

**Figure 1:**
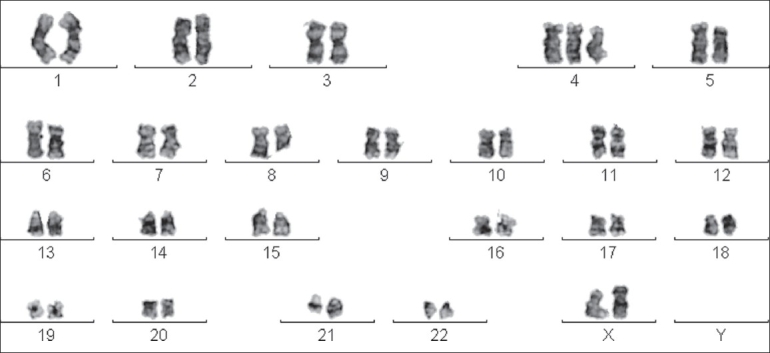
Cytogenetic result of unstimulated bone marrow samples showing 47,XX, +4, t(8;21) in all metaphase plates

Classical chromosome analysis detected an abnormal female chromosome complement. Karyotyping results revealed trisomy of 4 with t(8;21) in all metaphase plates in 15 metaphases [Figure 1].

### FISH analysis

**Figure 2:**
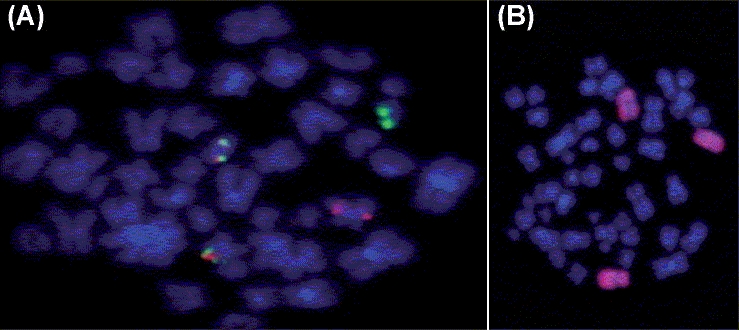
(A) A metaphase cell following FISH with LSI *AML-ETO* (Abbott Molecular, USA). (B) Whole chromosome paint probe 4 with spectrum Orange (Abbott Molecular, USA)

The interphase and metaphase FISH results were one green, one orange, and two green-orange or yellow fusion signals indicating *AML-ETO* fusion positive sample [Figure 2A]. The whole chromosome paint FISH confirmed trisomy of chromosome 4 with no other cryptic rearrangements [Figure 2B].

## Discussion

Trisomy 4 is a rare nonrandom cytogenetic abnormality found in association with AML. The Mitelman database for chromosomal aberrations in cancer queried for trisomy 4 with t(8;21) in AML showed only two cases.[6]

Gains or losses of chromosomes are frequent findings in AML, more common being monosomy 7 and trisomy 8. Most of such changes are not restricted to any specific FAB types of AML, are often also associated with secondary AML and AML with pre-existing myelodysplasia, or during clonal progression of AML. Rarely gain of chromosome 4 or chromosomes 10 are reported as the sole abnormality in AML.

Some morphologic subtypes of AML are associated with specific chromosomal abnormalities. Most of these abnormalities are chromosomal translocations which amplify or activate chimeric genes situated near the breakpoints of translocations. Trisomy 4 occurs in AML with frequency of <1% and a strong association with the presence of double minutes has been described. Double minutes were not observed in our patient.

CD56 expression in AML is reported in granulocytic sarcoma and multidrug resistance, and is known to confer poor prognosis in AML-M2 with t(8;21) and acute promyelocytic leukemia. It has been suggested that there might exist a dosage effect of certain genes resulting in growth advantage of malignant cells. In the case of AML with trisomy 4 and double minutes, V-myc myelocytomatosis viral oncogene homolog (avian) amplification is a common finding. Jennings *et al*, have also suggested that acquisition of trisomy 8 leading to MYC locus amplification might underline the molecular mechanisms for the clonal progression of chronic myeloid leukemia. A direct link between chromosomal gain, oncogenes amplification, and leukemogeneisis has yet to be established.[7,8]

Our patient was lost to follow up hence the CD56 status was unclear. Therefore, relation between CD56 expression and trisomy 4 needs further investigation. While development of AML with trisomy 4 secondary to chemo or radiotherapy has also been suggested, our patient had no history of long-term medication, radiotherapy, or any relevant occupational exposure. In conclusion, based on the morphological, cytochemistry, and clinical features the present case of AML-M2 is a rare case in terms of cytogenetic results. Even though trisomy 4 is likely to be a secondary event after t(8;21) translocation, the presence of this additional numberical aberration may define a distinctive subtype. Follow up of more such cases over a period of time is required to know the possible prognostic effect of this cytogenetic entity.[9]
